# P02 Doctor, I just don't feel alright

**DOI:** 10.1093/rap/rkab068.001

**Published:** 2021-10-19

**Authors:** Phing Sue Wong, Thomas Rhys, Mangat Pamela, Beynon Huw, Richard Stratton

**Affiliations:** University College London, London, United Kingdom

## Abstract

**Case report - Introduction:**

Antiphospholipid syndrome (APS), also known as Hughes syndrome, was first reported by Dr. Graham Hughes in 1983. It can be primary or secondary, with the latter associated with underlying rheumatological conditions. APS is an immune-mediated disorder with the hallmark of pregnancy morbidity and vascular thrombotic events associated with persistent antiphospholipid antibodies (aPLs). Rarely it causes catastrophic APS or Asherson syndrome. Neurological manifestation of APS is not uncommon.  It can affect central, peripheral as well as autonomic neural system through vascular thrombosis or autoimmune inflammatory-demyelination syndrome. Other relatively common clinical features livedo reticularis, thrombocytopenia, transient ischemic attack or movement disorders.

**Case report - Case description:**

Case 1

A 52-year-old Caucasian lady was seen in outpatient clinic for involuntary non-rhythmic movement of face, trunk and limbs and writhing of the fingers (athetosis).  Onset was 5 years ago and is progressing. She has Raynaud’s and livedo reticularis over her limbs.

She had two previous miscarriages and one pre-eclampsia.  Her immediate cousin has APS. Heart sounds were normal.

Platelet: 98 x10^9^/L

Two samples of high titre of ACA IgG, B2 Glycoprotein 1 Ab IgG, and positive Lupus anticoagulant 3 months apart.

Low complements C4

MRI brain showed T2/FLAIR signal within the supratentorial white matter.

3 weeks into treatments with steroid, hydroxychloroquine and VKA anticoagulation, patient reported improvement of symptoms.

Diagnosis: Choreo-athetosis in primary APLS

Case 2

A 42-year-old Caucasian female with chronic migraines was seen for imbalance gait and left upper limb compartment syndrome.

Vascular images showed left cerebellar ischemic infarct and left subclavian and brachial artery thrombosis without vasculitis or vascular malformation.

Patient had five obstretrics morbidities.

Echocardiogram was normal, aPLs were negative.

Patient underwent left arm fasciotomy. Immediate post operatively patient was anticoagulated and later switched to VKA and antiplatlet. She remains aPLs negative in outpatient visits.

Diagnosis: Non-criteria (seronegative) APLS with arterial thrombosis

Case 3

22-year-old, black female. Presented in 2018 with internal jugular, subclavian and pulmonary venous thrombosis. Her APTT was 38 and lupus anticoagulant were positive in two occasions 12 weeks apart. She received indefinite warfarin.

She reported persistent migraine headache. Brain imagings were insignificant. She refused lumbar puncture. Neuronal antibodies were negative.

Systemic glucocorticosteroid had improved her headache. Parenteral cyclophosphamide was started as she remained steroid refractory. Her headache resolved after completed course and reduced prednisolone dose.

She received 3.75 mg of leuprolide according to protocol.

Diagnosis: Refractory Migraine headache in Primary APS

**Case report - Discussion:**

Neurological manifestation for APLS is not uncommon. One review published the frequency of different neurological syndrome (Table 1).

Classically, aPLs target and bind to the phospholipid membranes of platelets with their subsequent activation and thrombosis. Recent evidence supports the concept of the mechanisms of immune-mediated vascular, inflammatory, and direct neurotoxic effects in neurological-APS manifestation. For instance, aPLs bind to endothelia, monocytes, neutrophils and interfere with complement activation. This results in immune-inflammatory response and coagulation cascade activation contributing to microcirculation thrombosis and ischemia. Combined procoagulation state, immune cascade activation plus aPL neurotoxicity, it further increases permeability of the blood—brain barrier with influx of inflammatory molecules into the nervous system.

There are currently 30 aPLs identified. LA, ACA and B2GP1A are studied most and are included in the classification criteria, hence also termed criterion aPL. Non-criterion aPLs, although lacking clinical representation, are getting more attention in investigating their relevance for the diagnosis, pathogenesis, and phenotype in APS. Examples are antibodies against aB2GPI (including IgA), Phosphatidylserine/prothrombin complex, Anti-phosphatidylserine (aPS), Anti-vimentin, Anti-phosphatidic acid, Anti-phosphatidylethanolamine (aPE). Four other aPLs shown to strongly correlate in APS-CNS manifestation are IgG antibodies against prothrombin (aPT), phosphatidylglycerol (aPG), phosphatidylinositol (aPI), and annexin-5 (aAN). Antibodies against aB2GPI (including IgA) were strongly associated with chorea in one publication. 66—88% of APS patients with migraine headache are positive to ACA.

**Case report - Key learning points:**

Sapporo classification criteria define definite APS as presence of one or more of clinical plus laboratory criteria. Clinical criteria include vascular thrombosis of venous, arterial, or small vessel and obstetric morbidity of early pregnancy loss or late pregnancy complications. Laboratory criteria are two positive results of either lupus anticoagulant, Anticardiolipin or Anti-β2-glycoprotein-I IgG or IgM (ELISA) in significant titre 12 weeks apart.

Although APS patients can present with young stroke, other differentials are vascular malformation and arteriopathy (eg. Moya-moya and CADASIL), systemic infections, cardiac, metabolic disorders (e.g., ADA deficiency, Fabry’s disease, Homocystinuria), sickle cell disease, inherited prothrombotic conditions, paroxysmal nocturnal haemoglobinuria and substances abuse.

APS management is largely based on serological risk stratification. Presence of Lupus anticoagulant with or without persistent ACA and B2GPA in high titre are considered high-risk group.

Low-dose aspirin (LDA, 75-100mg) used in primary prophylaxis reduces half of thrombotic events. Addition of prophylaxis dose of heparin is recommended for all pregnant females with history of obstetrics APS.

Anticoagulation therapy with vitamin-K antagonist (VKA) is the main strategy in secondary thromboprophylaxis in APS. Therapeutic target of INR varies. INR of 2–3 is the target in venous thrombosis, in arterial thrombosis INR should target at 2—4 based on bleeding risk in addition to LDA.   Higher INR is also indicated in refractory cases, as well as addition of LDA or switch to LMWH. Patients of high-risk groups and with recurrent events should receive indefinite anticoagulant.

Several major trials of DOACs failed to reach primary efficacy and safety endpoints, in particular rivaroxaban, hence these should be avoided particularly in arterial thrombosis.

Evidence with immunosuppressants remain controversial; EULAR recommended HCQ and prednisolone in refractory obstetric APS. Immunoglobulin, plasma exchange, rituximab and eculizumab have been reported successfully used in CAPS, isolated cases as well as refractory APS.

**Table:** 

Neurological Manifestation	Frequency (%)
Cerebral Venous Thrombosis	0.7%
Ischemic Stroke	20%
TIA	11%
Sneddon’s syndrome	-
Seizures and Epilepsy	6 to 9%
Cognitive and Neuropsychiatric Manifestations	42– 80%
Migraine headache	20%
Transverse Myelopathy and Myelitis	0.4–4%
Movement disorders Chorea Ballismus Dyskinesia, parkinsonism, and , cerebellar ataxia	1% 0.3% -
Peripheral Nervous System mononeuritis multiplex presentation polyneuropathy distal axonal sensorimotor neuropathy immune-mediated peripheral nerve syndromes (eg. Guillain–Barré syndrome)	Eg. carpal tunnel syndrome (23%) 19% 15% -
Autonomic disorders	-
Demyelination disorders	-

